# Emerging treatments for neuromyelitis optica spectrum disorder

**DOI:** 10.1007/s00415-020-10083-3

**Published:** 2020-07-22

**Authors:** Ray Wynford-Thomas, Neil P. Robertson

**Affiliations:** Institute of Psychological Medicine and Clinical Neuroscience, Cardiff University, University Hospital of Wales, Heath Park, Cardiff, CF14 4XN UK

## Introduction

Neuromyelitis optica spectrum disorder (NMO-SD) has a worldwide prevalence of 0.5–10 persons per 100,000 population. It is characterised by inflammatory events centred on the optic nerve and spinal cord, often with poor recovery and subsequent residual disability. More than two-thirds of patients with NMO-SD have serum antibodies to the water channel protein aquaporin-4 (AQP4-immunoglobulin G [IgG]), which is implicated in the pathophysiology of NMO-SD. Others are seronegative or have serum antibodies to myelin oligodendrocyte glycoprotein. Diagnostic criteria depend on AQP4-IgG antibody status: those with seropositivity can have involvement of almost any CNS region, whereas those who are seronegative must have experienced at least one of either an optic neuritis, longitudinally extensive transverse myelitis, or area postrema syndrome with associated MRI lesions. Treatment of NMO-SD to date has largely been based on observational studies, case reports and retrospective analyses, and has included empiric use of off-label treatments such as rituximab, azathioprine and mycophenolate mofetil. However, with the recent completion of phase 3 clinical trials, an improved evidence base for current treatments and novel treatment options for NMO-SD are now emerging. Three clinical trials evaluate the monoclonal antibodies rituximab, satralizumab and eculizumab, versus placebo in the treatment of NMO-SD, and are discussed below.

## Safety and efficacy of rituximab in neuromyelitis optica spectrum disorders (RIN-1 study): a multicentre, randomised, double-blind, placebo-controlled trial

Rituximab targets CD20 (a surface antigen primarily expressed on B lymphocytes) and causes B cell depletion. In this trial, NMO-SD patients with current or previous AQP4 seropositivity, and an Expanded Disability Status Scale (EDSS) score of 7.0 or less, were randomly assigned (1:1) treatment with rituximab (intravenous [IV], every week for 4-weeks and then 6-month interval dosing; *n* = 19) or placebo (matched administration; *n* = 19). To reduce excessive risk of relapse, patients were also treated with 2-months of a fixed dose of oral steroid, which was then slowly reduced. The primary outcome was time to first relapse in the 72-week study period. No relapses occurred in the rituximab group, whereas seven (37%) relapses occurred in the placebo group (*p* = 0.0058). The EDSS score worsened in those who had relapses; however, the average change in EDSS score was similar between the two study groups. Conversely, the authors report that the average change in total quantification of optic nerve and spinal cord impairment (QOSI) score was significantly higher in the placebo group. 37% of patients had an infusion reaction in the rituximab group, compared to none in the placebo group (*p* = 0.008). Otherwise adverse events did not differ significantly between the groups. One patient in the rituximab group discontinued secondary to an adverse event. There were no cases of progressive multifocal leukoencephalopathy.

*Comment.* As one might have expected, this trial reports positive findings for the use of rituximab for relapse prevention in NMO-SD patients who are AQP4 seropositive. A limitation of this trial is the small sample size, which the authors state does not allow for an accurate quantification of the magnitude of risk reduction attributed to rituximab. Furthermore, the study only included patients who were adults, of Japanese ethnic origin, and who had AQP4 seropositivity, limiting the generalisability of the results. The power of the trial may have been reduced by the use of concomitant steroids, and the difference in annualised relapse rate (ARR) in the 2 years pre-enrolment between the rituximab group (1.4 relapses per person-year) and the placebo group (0.7 relapses per person-year), could have resulted in an underestimation of the efficacy of rituximab. Despite its efficacy in relapse prevention, it is interesting that there was no significant change in the EDSS score between both groups, possibly due to the use of concomitant steroids. They did report a significant change in the QOSI; however, this score is not widely used and its psychometric properties have not been studied in NMO-SD. Larger trials would clearly be of value and further studies are warranted to assess the efficacy of rituximab in AQP4 seronegative patients.

Tahara M et al. (2020) Safety and efficacy of rituximab in neuromyelitis optica spectrum disorders (RIN-1 study): a multicentre, randomised, double-blind, placebo-controlled trial. Lancet Neurol 19(4):298–306.

## Safety and efficacy of satralizumab monotherapy in neuromyelitis optica spectrum disorder: a randomised, double-blind, multicentre, placebo-controlled phase 3 trial

Satralizumab binds to IL­6 receptors, inhibiting the IL­6 signalling pathways involved in inflammation. In this trial, patients with AQP4-positive or AQP4-negative NMO-SD, and an EDSS score of 6.5 or less, were randomly assigned (2:1) treatment with satralizumab (subcutaneous, every 2-weeks for 4 weeks and every 4-weeks thereafter; *n* = 63) or placebo (matched administration; *n* = 32). Steroid use was only allowed for the acute treatment of relapses. As concomitant immunosuppressants were prohibited, unequal randomisation was used to minimise risk of harm of no treatment. The primary outcome was time to first protocol-defined relapse in the double-blind period (1·5 years after the random assignment of the last enrolled patient). 30% of the satralizumab group had a protocol-defined relapse, compared to 50% of the placebo group (*p* = 0.018). Furthermore, those in the placebo group had a shorter time to relapse. However, when analysed by AQP4 serostatus, the results differed. In the seropositive cohort, 22% in the satralizumab group had a protocol-defined relapse, versus 57% in the placebo group. Conversely in the seronegative cohort, 46% in the satralizumab group experienced a protocol-defined relapse, versus 33% in the placebo group. For all patients, mean change in EDSS score at week 24 was − 0·34 for the satralizumab group and − 0·17 for the placebo group. The overall rate of infections was similar between the two groups, and no opportunistic infections were reported in those treated with satralizumab. Severe adverse events were higher in the satralizumab group (32·1 events per 100 patient-years; versus 9·9 events per 100 patient-years in the placebo group). The authors state that 73% were considered unrelated to the study treatment, however one (pneumonia) led to discontinuation of the study drug.

*Comment.* This trial demonstrates encouraging results for the use of satralizumab in relapse prevention for patients with NMO-SD and AQP4 seropositivity. The evidence however was insufficient to support use for seronegative patients. In contrast to the rituximab trial, patients in this trial were only eligible if they had a clinical relapse in the last 12-months, thereby potentially inherently selecting those with a higher disease activity. Furthermore, the prohibition of concomitant treatments allowed for more definite clarification of the effect of satralizumab in the treatment of NMO-SD. Other limitations of this trial include small group numbers, low relapse numbers, and inclusion of adults only.

Traboulsee A et al. (2020) Safety and efficacy of satralizumab monotherapy in neuromyelitis optica spectrum disorder: a randomised, double-blind, multicentre, placebo-controlled phase 3 trial. Lancet Neurol 19(5):402–412.

## Eculizumab in Aquaporin-4–positive neuromyelitis optica spectrum disorder

Eculizumab inhibits the terminal complement protein C5, impeding the complement cascade at this point. In this phase 3 double-blind trial, patients with NMO-SD and AQP4 seropositivity, with an EDSS score of 7.0 or less, were randomly assigned (2:1) treatment with eculizumab (IV, every week for the first four doses, followed by every 2-weeks; *n* = 96) or placebo (matched administration; *n* = 47). If patients were already receiving immunosuppressive therapies for relapse prevention, they were still eligible for inclusion, as long as they were on a stable drug regimen; however, this excluded treatment with rituximab or mitoxantrone in the last 3-months, prednisone in doses greater than 20 mg/day or equivalent and IVIg in the previous 3-weeks. Crucially, the study also excluded anyone with unresolved meningococcal disease, or other infections considered to be clinically significant or not treated with appropriate antibiotics, and patients received a vaccination against Neisseria meningitidis before receiving a study agent. This is because eculizumab increases the risk of infection with encapsulated organisms, and fatalities from meningococcal infections have previously been reported. The primary end point of first adjudicated relapse occurred in 3% of the eculizumab group and 43% of the placebo group (*p* < 0.001). In those not receiving concomitant immunosuppressive therapy (approximately one-quarter of patients), no adjudicated relapses occurred in the eculizumab group (*n* = 21), whereas 54% occurred in the placebo group (*n* = 13). There was a significantly lower adjudicated ARR in the eculizumab group compared to the placebo group. The difference between groups in change in EDSS score was not significant. In the eculizumab group, rates of upper respiratory tract infection were higher; furthermore, one patient, who was also receiving azathioprine, died from a pulmonary empyema which was deemed probably related to the trial agent. No cases of meningococcal infection were reported, and no patients discontinued eculizumab due to adverse events.

*Comment*. Finally, this trial similarly shows promising results for the use of eculizumab in relapse prevention in NMO-SD AQP4-positive patients, although it found no significant between-group difference in measures of disability progression. Again, in contrast to the previous trials, it should be noted that patients were only eligible for inclusion if they had at least two relapses in the last 12-months, or three relapses in the previous 24-months, one of which had to occur in the previous year, raising the possibility of selection bias for those with higher disease activity. Additionally, it is important to highlight that each trial used different criteria to define a relapse; in particular the rituximab study required objective abnormalities on MRI. The death of a study participant in the eculizumab trial is an important reminder of the risks that immunosuppressive treatments carry. Limitations of this trial include the exclusion of AQP4 seronegative patients and the inclusion of adults only. Furthermore, the power of the trial was blunted due to earlier termination than originally planned, and the trial allowed the use of concomitant immunosuppressive therapies in around three-quarters of patients.

Pittock SJ et al. (2019) Eculizumab in aquaporin-4–positive neuromyelitis optica spectrum disorder. N Engl J Med 381(7):614–625.

